# Multivariate evaluation of protein and energy utilization in Peruvian Guinea pigs (*Cavia porcellus*) under different feeding regimens

**DOI:** 10.14202/vetworld.2025.2774-2784

**Published:** 2025-09-18

**Authors:** William Armando Tapie, Carlos Santiago Escobar-Restrepo, Juan Fernando Manrique-Hincapie

**Affiliations:** 1 GIAZ Research Group, Faculty of Agricultural Sciences, Catholic University of the East (UCO-Universidad Católica de Oriente), AA 008, Rionegro, Antioquia, Colombia; 2 INCA-CES Research Group, Faculty of Veterinary Medicine and Animal Husbandry, University CES (Universidad CES), Cl 10A #22 - 04, Medellín, Antioquia, Colombia

**Keywords:** crude protein, guinea pig, metabolizable energy, nutrient efficiency, principal component analysis

## Abstract

**Background and Aim:**

Guinea pig (*Cavia porcellus*) production is vital for food security in Andean countries and increasingly relevant in parts of Africa. Optimizing nutrient utilization is critical to enhance productivity, farmer income, and sustainability. This study employed a multivariate approach to evaluate crude protein and energy digestibility and metabolism in Peruvian guinea pigs under different feeding regimens (maintenance, restricted, and *ad libitum*) at various ages.

**Materials and Methods:**

Forty-two male guinea pigs were housed individually in metabolic cages and fed a pelleted diet formulated according to the National Research Council (1995) recommendations. Digestibility and metabolism trials were conducted at 52, 90, and 145 days of age across three feeding levels. Variables including dry matter intake (DMI), gross energy intake (GEI), digestible energy, metabolizable energy (ME), crude protein intake (CPI), and retained protein (RP) were measured. Data were analyzed using principal component analysis (PCA) and hierarchical clustering to identify intake-efficiency patterns.

**Results:**

DMI, GEI, and CPI increased significantly with age and feeding level, strongly correlating with body weight (r > 0.7). Protein retention efficiency (RP/CPI) was highest at maintenance feeding (83.5%) but declined to 73.6% in *ad libitum*-fed animals at 145 days, indicating protein catabolism when intake exceeded requirements. In contrast, energy metabolizability (ME/GE) peaked under *ad libitum* feeding (79.5% at 90 days). PCA revealed that PC1 (48.5% variance) was associated with intake and growth, whereas PC2 (18.1%) was linked to metabolic efficiency of protein and energy. Cluster analysis distinguished three groups by feeding level and age, confirming that higher intake reduced protein utilization efficiency despite supporting faster growth.

**Conclusion:**

Multivariate analysis demonstrated that while *ad libitum* feeding maximized growth and energy metabolizability, it reduced protein retention efficiency, emphasizing the need for balanced protein–energy ratios tailored to the physiological stage. These findings provide a framework for designing age- and intake-specific feeding strategies to enhance nutrient efficiency, meat production, and sustainability in guinea pig systems.

## INTRODUCTION

Guinea pig (*Cavia porcellus*) production plays a vital role in food security across Andean countries, such as Peru, Bolivia, Colombia, and Ecuador, and has been incorporated into diets in sub-Saharan Africa for several decades [1, 2]. Strengthening production systems is increasingly essential to raise farmer incomes and promote rural development [3, 4]. Achieving this goal requires the adoption of genetically improved lines with higher productivity than traditional native breeds [2], alongside nutritionally balanced feeding strategies.

The development of cost-effective diets depends on accurate knowledge of the nutritional value of feedstuffs [1] and the specific nutrient requirements of guinea pigs [5]. While several studies have assessed the nutritional contribution of feeds and determined energy and protein needs for meat production [1, 4, 6–8], there remains limited information on how age and feeding level interact to influence these requirements [9]. Factors such as breed, age, sex, and environmental conditions affect body size, which in turn influences energy and protein demand [10]. Feed utilization efficiency in guinea pigs is determined by diet composition, energy concentration, and the animals’ ability to digest and metabolize nutrients [11]. Previous studies on energy and protein requirements, digestibility, and metabolism in guinea pigs [4, 8] often overlooked critical aspects such as nutrient interactions, metabolic losses (fecal, urinary, and heat), feeding intensity, and age-related changes. Because carcass composition (fat and protein content) shifts with age and growth [4, 12], digestibility patterns also change, directly impacting dietary needs. Unlike other species, reduced dry matter intake (DMI) in guinea pigs is linked to longer gastrointestinal retention time, which increases enzymatic exposure and enhances nutrient digestibility [13]. However, higher fecal output does not necessarily indicate improved digestibility or energy metabolizability, and restricted feed intake often increases urinary energy losses [4]. Similarly, protein digestibility (PD) and retention efficiency decrease as intake levels rise, although urinary protein losses remain relatively stable across diets [8].

Given the complex interplay among DMI, dietary energy, protein intake, and excretory losses, multivariate approaches such as principal component analysis (PCA) provide a more comprehensive understanding by reducing data dimensionality. This method enables the joint evaluation of multiple variables rather than considering them individually [14]. Integrating such analyses allows a clearer interpretation of digestive and metabolic processes across feeding levels and ages, supporting the design of optimized nutritional programs, improving productivity, and enhancing the efficiency of feed resource utilization in guinea pig meat production systems [5].

Despite the growing importance of guinea pig (*C. porcellus*) production for food security and rural livelihoods in Andean countries and parts of Africa, knowledge about their nutritional physiology remains limited compared with other livestock species. Existing studies have primarily focused on general nutrient requirements and feedstuff evaluation for energy and protein supply [1, 4, 6–8]. However, few have systematically examined how feeding intensity and animal age jointly influence protein and energy digestibility, metabolizability, and retention efficiency. Prior research has also tended to employ univariate approaches, which fail to capture the complex interactions among nutrient intake, metabolic losses (fecal, urinary, and heat), and growth stage. Moreover, although carcass composition and nutrient utilization are known to shift with age [4, 12], there is insufficient information on how these dynamics affect optimal feeding strategies in meat production systems. A further limitation is that protein retention efficiency often declines under higher intake levels, but the underlying metabolic relationships have not been fully explored. This lack of integrated analysis constrains the development of precise, cost-effective, and environmentally sustainable feeding programs tailored to the unique physiology of guinea pigs.

To address these gaps, the present study applied a multivariate approach–specifically PCA and hierarchical clustering–to evaluate the digestibility and metabolism of crude protein and energy in Peruvian breed guinea pigs. The research examined animals under three feeding regimens (maintenance, restricted, and *ad libitum*) at three age stages (52, 90, and 145 days) to identify key patterns of intake, utilization efficiency, and nutrient partitioning. By integrating multiple metabolic variables into a holistic framework, this study aimed to: (i) reveal how age and feeding level interact to influence nutrient use efficiency; (ii) distinguish metabolic clusters associated with growth and intake levels; and (iii) generate evidence to guide the formulation of optimized, stage-specific diets. Ultimately, the findings are expected to provide a foundation for designing feeding strategies that improve production efficiency, enhance farmer profitability, and reduce nitrogen-related environmental impacts in guinea pig production systems.

## MATERIALS AND METHODS

### Ethical approval

The experiment was approved by the Ethics Committee for Animal Experimentation of the University of Antioquia (Act No. 138, February 9, 2021). All procedures followed the guidelines established in the *Animal Research: Reporting of in vivo Experiments* (ARRIVE 1.0) framework.

### Study period and location

The study was conducted from February 2021 to September 2023 at the Santa María Center of the Catholic University of the East, located in the municipality of El Carmen de Viboral (6°04’23.2”N 75°22’44.5”W), Colombia.

### Animals and housing

A total of 42 male Peruvian breed guinea pigs were obtained from the Minor Species Production Unit of the International Clean Production Center Lope - National Learning Service, Colombia. At the time of purchase, animals had an average live weight (LW) of 393 ± 55 g and were 35 days old. A 20-day acclimatization period was provided to familiarize the animals with handling and the experimental diet before data collection. During the study, guinea pigs were individually housed in metabolic cages (0.30 × 0.30 × 0.25 m), each equipped with an automatic feeder and water dispenser. Housing conditions were controlled to maintain ambient temperature between 18°C and 22°C and relative humidity between 65% and 75%.

### Diet composition

The basal diet was formulated according to the recommendations of the National Research Council [15]. Due to ingredient availability, wheat (23.6%) and whole oats (25.2%) were replaced with rice flour and corn (Table 1). Feed was pelleted and offered twice daily (07:00 and 15:00 h). Daily feed intake was determined by subtracting refusals from the total amount of feed offered.

**Table 1:** Ingredients and chemical composition of the experimental diet (dry matter basis) for guinea pigs.

Ingredients	Percentage
Alfalfa hay	35.0
Soybean meal, expeller	12.0
Corn	44.3
Rice flour	3.0
Soybean oil	3.0
Dicalcium phosphate	0.5
Calcium carbonate	1.0
Salt	0.8
Mineral premixes and vitamins^1^	0.4
Chemical composition	
Dry matter (%)	92.1
Crude protein (%)	16.4
Ether extract (%)	4.0
Ash (%)	5.5
NDF (%)	22
ADF (%)	16
Non-fiber carbohydrates^2^ (%)	50.5
Digestible energy (kcal/kg DM)	3,705
Metabolizable energy (kcal/kg DM)	3,520

1 Minerals: Cobalt 1.5; copper 6.6; manganese 39.7; zinc 19.8; iodine 1.1; iron 50; selenium 0.3 (mg kg^−1^). Vitamins: Vitamin A 6614; vitamin D3 2200 (IU kg^−1^), vitamin E 22; vitamin K 5; thiamine 4.4; riboflavin 3.3; niacin 11; pantothenic acid 11; choline 529; pyridoxine 5; folic acid 4.8; biotin 2.2; ascorbic acid 250; methionine hydroxy analog 500 (mg kg^−1^); vitamin B12 11 μg kg^−1^. Antioxidant BHT 0.1 g kg^−1^; Salinomycin 20 mg kg^−1^.

2 NFC = 100% - (Crude protein + Ether extract + Ash + Neutral detergent fiber), kcal/kg = Kilocalories/kilogram, DM = Dry matter

### Feeding regimens

Digestibility trials were carried out at three age stages (52, 90, and 145 days) and under three feeding levels: maintenance, restricted, and *ad libitum*. Each trial lasted 6 days, following the methodology of Castro *et al*. [7] and Tapie *et al*. [4]. Trials were conducted independently for each age group.

- At 52 days of age, 12 animals were maintained at a feeding level of 115.2 kcal digestible energy (DE)/kg body weight (BW)^0.75^ [16].
- At 90 days, animals were assigned to three groups (n = 6 per group): maintenance, restricted, and *ad libitum*. The *ad libitum* group received feed to allow ~20% refusals, while the restricted group was fed at 75% of *ad libitum* intake.
- At 145 days, only *ad libitum* and restricted groups (n = 6 each) were evaluated.

At the end of the final trial, animals were humanely slaughtered using a Dick KTBG captive bolt gun (Friedr Dick GmbH and Co., Deizisau, Germany), following the protocol described by Limon *et al*. [17].

### Sample collection

DMI was calculated as the difference between feed offered and refusals. Feces and refusals were collected daily, weighed, and pooled for each animal. Urine was collected in containers containing 5 mL of 5% sulfuric acid to prevent nitrogen volatilization. Daily urine volume and weight were measured, and composite samples were prepared for each guinea pig. All samples of feces, urine, and feed were stored at –15°C until laboratory analysis.

### Laboratory analysis

Feed, refusals, feces, and urine were analyzed for:

- Dry matter (gravimetric method [18]),
- Crude protein (Kjeldahl method [18]), and
- Gross energy (GE) (LECO AC600 bomb calorimeter, MI, USA).

### Calculations of energy and protein balance

Nutrient intake was determined as:

(DM, CP, and GE) Intake = (DM, CP, and GE) feed – (DM, CP, and GE) refusals (DM, CP) (1)

Digestibility (%) was calculated as:


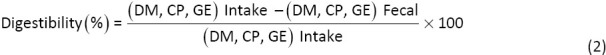


Metabolizable energy intake (MEI) was estimated as:


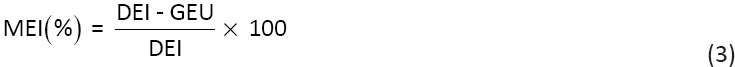


Retained protein (RP), representing net protein balance, was calculated as:

RP = CPI – (CPF + CPU) (4)

Where:

- DM = Dry matter (g/d),
- CP = Crude protein (g/d),
- GE = Gross energy (kcal/d),
- DEI = DE intake (kcal/d),
- GEU = Gross energy lost in urine (kcal/d),
- CPI = Crude protein intake (g/d),
- CPF = Crude protein lost in feces (g/d),
- CPU = Crude protein lost in urine (g/d).

### Statistical analysis

Data were analyzed using PCA with the *FactoMineR* package (version 2.11) in R software (version 4.4.2; R Core Team, R Foundation for Statistical Computing, Vienna, Austria) [19]. Variables were standardized before analysis. Principal components were selected using two complementary criteria: (i) The Kaiser criterion, which retains components with eigenvalues above the mean [14, 20], and (ii) cumulative variance explained by the first two components. Hierarchical clustering was performed using the unweighted pair group method with arithmetic mean and Euclidean distances. Graphical outputs, including PCA biplots and heatmaps, were generated with the ggplot2 (version 3.4.4) and *complo*t packages [19].

## RESULTS

### Energy and protein intake and digestibility

Table 2 presents the mean values and standard deviations for energy and crude protein intake (CPI), digestibility, and metabolism in guinea pigs evaluated at three ages (52, 90, and 145 days) under three feeding levels (maintenance, restricted, and *ad libitum*). BW varied notably across feeding levels, ranging from 396.1 g in the maintenance group to 1142.9 g in the *ad libitum* group. Dry matter digestibility (DMD) was highest in the maintenance group (82.1%–82.3%) compared with those fed *ad libitum* (78.8%–81.2%). Similarly, the ratio of DE to GE was greater in maintenance-fed animals (81.5%–82.1%) than in those receiving *ad libitum* diets (78.8%–81.2%). PD relative to CPI (PD/CPI) followed the same trend, ranging from 81.3% to 84.5% in the maintenance group versus 75.4% in the *ad libitum* group.

**Table 2:** Energy and crude protein partitioning (dry matter basis) for three feeding levels in Peruvian guinea pigs.

Parameter	Maintenance		Restricted		*ad libitum*	
Age (d)	52.0 ± 3.46	90.0 ± 3.35	90 ± 1.50	145 ± 1.50	90 ± 2.66	145 ± 2.66
Body weight (g)	396.1 ± 49.64	481.8 ± 60.68	611.0 ± 81.15	870.0 ± 173.32	698.2 ± 34.56	1142.9 ± 119.86
DMI (g/d)	21.9 ± 2.74	23.2 ± 2.34	37.2 ± 5.04	40.1 ± 7.98	42.9 ± 3.55	48.7 ± 5.07
DMI (%BW)	5.5 ± 0.00	4.8 ± 0.29	6.09 ± 0.15	4.6 ± 0.00	6.1 ± 0.39	4.3 ± 0.16
DMD (%)	82.1 ± 3.50	82.3 ± 1.72	80.6 ± 1.76	80.5 ± 1.03	80.9 ± 1.21	78.1 ± 6.51
Energy balance (kcal/d)						
GEI	98.8 ± 12.39	104.7 ± 10.54	168.0 ± 22.76	180.9 ± 36.04	196.7 ± 16.90	222.1 ± 23.35
Fecal DM (g/d)	3.9 ± 0.91	4.1 ± 0.67	7.2 ± 0.93	7.9 ± 1.91	8.2 ± 0.94	10.8 ± 3.87
Fecal GE	17.7 ± 4.30	19.4 ± 3.20	32.5 ± 3.80	34.9 ± 9.00	37 ± 4.20	47.5 ± 16.00
DEI	81.1 ± 10.75	85.3 ± 8.45	135.5 ± 20.14	146.0 ± 27.04	159.7 ± 13.28	174.6 ± 19.74
Urinary DM (g/d)	2.4 ± 0.52	1.9 ± 0.30	2.4 ± 0.48	2.5 ± 0.98	2.0 ± 1.23	2.8 ± 0.59
UE	4.9 ± 1.75	4.6 ± 1.14	5.8 ± 1.97	5.3 ± 2.40	3.4 ± 1.74	5.3 ± 1.94
MEI	76.2 ± 10.60	80.7 ± 9.21	129.6 ± 21.41	140.7 ± 26.72	156.3 ± 12.32	169.3 ± 20.27
Energy utilization efficiency (%)					
DEI/GEI	82.1 ± 3.67	81.5 ± 2.12	80.5 ± 1.78	80.9 ± 1.30	81.2 ± 1.09	78.8 ± 5.92
MEI/GEI	77.1 ± 4.10	77.0 ± 2.77	76.9 ± 3.01	77.9 ± 1.17	79.5 ± 1.60	76.4 ± 6.60
MEI/DEI	94.0 ± 2.10	94.5 ± 1.61	95.5 ± 2.00	96.3 ± 1.64	97.9 ± 0.96	96.9 ± 1.32
Fecal GE/GEI	18.00 ± 3.67	18.5 ± 2.12	19.5 ± 1.78	19.1 ± 1.30	18.8 ± 1.09	21.2 ± 5.92
UE/GEI	5.0 ± 1.71	4.5 ± 1.28	3.6 ± 1.55	3.0 ± 1.35	1.7 ± 0.77	2.4 ± 0.78
Protein balance (g/d)						
CPI	3.6 ± 0.45	3.8 ± 0.38	6.1 ± 0.82	6.6 ± 1.31	6.9 ± 0.65	8.10 ± 0.81
Fecal CP	0.7 ± 0.15	0.6 ± 0.09	1.1 ± 0.18	1.3 ± 0.35	1.7 ± 0.23	2.0 ± 0.65
DP	2.9 ± 0.41	3.2 ± 0.31	5.0 ± 0.71	5.2 ± 0.97	5.2 ± 0.55	6.1 ± 0.73
Urinary CP	0.09 ± 0.03	0.04 ± 0.01	0.06 ± 0.02	0.09 ± 0.03	0.1 ± 0.09	0.15 ± 0.07
RP	2.83 ± 0.41	3.2 ± 0.32	4.9 ± 0.72	5.1 ± 0.96	5.1 ± 0.47	6.0 ± 0.76
Protein utilization efficiency (%)						
DP/CPI	81.3 ± 3.90	84.5 ± 1.30	81.9 ± 2.30	79.9 ± 1.70	75.4 ± 2.50	75.4 ± 6.70
RP/CPI	78.8 ± 3.90	83.5 ± 1.30	80.9 ± 2.60	78.4 ± 1.60	74.1 ± 2.10	73.6 ± 7.30
RP/DP	96.9 ± 1.20	98.8 ± 0.30	98.8 ± 0.40	98.1 ± 0.30	98.2 ± 1.30	97.4 ± 1.30
Urinary CP/DP	3.0 ± 1.22	1.1 ± 0.31	1.2 ± 0.46	1.8 ± 0.38	1.7 ± 1.32	2.6 ± 1.33

DMD = Dry matter digestibility, GEI = Gross energy intake, GE = Gross energy, DM = Dry matter, DEI = Digestible energy intake, MEI = Metabolizable energy intake, CPI = Crude protein intake, CP = Crude protein, DP = Digestible protein, UE = Urinary energy, RP = Retained protein, BW = Body weight, ± = Standard deviation, g/d = Grams/day, d = Day, kcal = Kilocalories

### Correlation analysis

**Figure 1:**
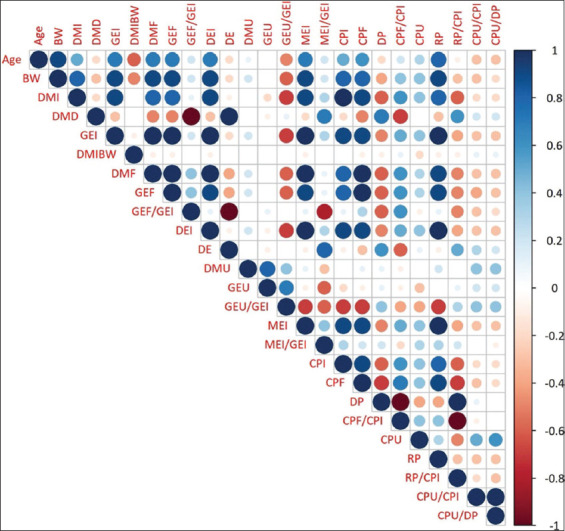
Correlation heatmap of energy and crude protein digestibility variables in Peruvian guinea pigs. The intensity of the blue color represents positive correlations, and the intensity of the red color represents negative correlations. BW = Body weight, DMI = Dry matter intake, DMD = Dry matter digestibility, GEI = Gross energy intake, DMI = Dry matter intake, DMF = Fecal dry matter, GEF = Fecal gross energy, GEF/GEI = Ratio of fecal energy to gross energy intake, DEI = Digestible energy intake, DE = Digestible energy, DMU = Urinary dry matter, GEU = Urinary gross energy, GEU/GEI = Ratio of urinary energy to gross energy intake, MEI = Metabolizable energy intake, MEI/GEI = Energy metabolizability, CPI = Crude protein intake, CPF = Fecal crude protein, DP = Digestible protein, CPF/CPI = Ratio of fecal crude protein to crude protein intake, CPU = Urinary crude protein, RP = Retained protein, RP/CPI = Protein retention efficiency, CPU/CPI = Ratio of urinary protein to crude protein intake, CPU/DP = Ratio of urinary protein to digestible protein.

Figure 1 illustrates the correlations among energy and crude PD variables in the form of a heatmap. The circle size and color intensity represent the magnitude and direction of each correlation. Strong positive correlations (r > 0.7) were observed between BW and absolute intake variables, including DMI, GE intake (GEI), MEI, CPI, and RP. These variables also showed strong associations with fecal excretion of energy and protein.

### PCA

Table 3 shows the factor loadings from the PCA applied to digestibility and metabolism variables. Although the first five principal components had eigenvalues >1 and accounted for more than 5.89% of the variance, PC1 (48.51%) and PC2 (18.13%) captured the largest proportion of total variance. The variables contributing most strongly to PC1 were fecal dry matter, fecal GE (GEF), and fecal crude protein (CPF), with loadings of 0.280, 0.279, and 0.283, respectively. In contrast, PC2 was primarily defined by MEI relative to GEI (MEI/GEI) and DE, with loadings of 0.438 and 0.400, respectively.

**Table 3:** Principal components of the energy and crude protein digestibility variables.

Parameter	PC1	PC2	PC3	PC4	PC5
Age	0.204	0.097	0.100	−0.227	−0.366
BW	0.255	0.100	−0.001	−0.178	−0.216
DMI	0.263	0.083	−0.080	0.064	0.131
DMD	−0.140	0.384	−0.114	−0.018	0.092
GEI	0.276	0.083	−0.004	−0.098	0.087
DMI/BW	−0.039	−0.077	−0.063	0.208	0.702
Fecal DM	0.280	−0.017	0.029	−0.095	0.051
Fecal GE	0.279	−0.024	0.041	−0.096	0.053
Fecal GE/GEI	0.102	−0.400	0.163	0.006	−0.113
DEI	0.272	0.110	−0.015	−0.097	0.094
DE	−0.102	0.400	−0.163	−0.006	0.113
Urinary DM	0.015	−0.071	−0.253	−0.499	0.217
Urinary GE	−0.040	−0.170	−0.086	−0.523	0.190
Urinary GE/GEI	−0.206	−0.188	−0.098	−0.272	0.067
MEI	0.273	0.117	−0.011	−0.074	0.086
MEI/GEI	0.045	0.438	−0.070	0.163	0.050
CPI	0.263	0.083	−0.080	0.064	0.131
Fecal CP	0.283	−0.007	−0.055	0.012	0.072
DP	−0.198	0.240	0.128	−0.226	−0.041
Fecal CP/CPI	0.198	−0.240	−0.128	0.226	0.041
Urinary CP	0.117	0.059	−0.401	0.153	−0.319
RP	0.263	0.115	0.029	−0.159	0.066
RP/CPI	−0.173	0.245	0.252	−0.213	−0.015
Urinary CP/CPI	−0.094	−0.034	−0.525	−0.043	−0.107
Urinary CP/DP	−0.079	−0.068	−0.531	−0.028	−0.115
Eigenvalue	12.130	4.530	3.000	2.640	1.470
Variance	48.510	18.130	11.990	10.570	5.890

BW = Body weight, DMI = Dry matter intake, DMD = Dry matter digestibility, GEI = Gross energy intake, DM = Dry matter, DEI = Digestible energy intake, MEI = Metabolic energy intake, DE = Digestible energy, GE = Gross energy, ME = Metabolic energy, CPI = Crude protein intake, CP = Crude protein, DP = Digestible protein, RP = Retained protein

### PCA clustering of feeding levels and age groups

**Figure 2:**
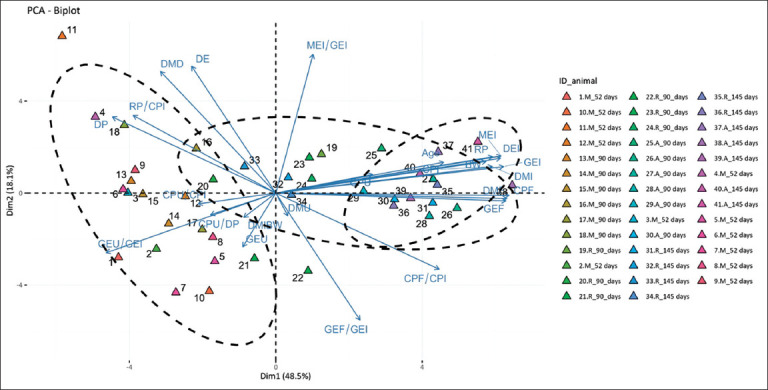
Clustering of energy and crude protein digestibility variables, feeding levels, and age in Peruvian guinea pigs. The vectors’ directions represent the variable’s contribution to each PCA, and the dotted circles represent groups of animals with similar characteristics. BW = Body weight, DMI = Dry matter intake, DMD = Dry matter digestibility, GEI = Gross energy intake, DMF = Fecal dry matter, GEF = Fecal gross energy, GEF/GEI = Ratio of fecal energy to gross energy intake, DEI = Digestible energy intake, DE = Digestible energy, DMU = Urinary dry matter, GEU = Urinary gross energy, GEU/GEI = Ratio of urinary energy to gross energy intake, MEI = Metabolizable energy intake, MEI/GEI = Energy metabolizability; CPI = Crude protein intake, CPF = Fecal crude protein, DP = Digestible protein, CPF/CPI = Ratio of fecal crude protein to crude protein intake, CPU = Urinary crude protein, RP = Retained protein, RP/CPI = Protein retention efficiency, CPU/CPI = Ratio of urinary protein to crude protein intake, CPU/DP = Ratio of urinary protein to digestible protein.

Figure 2 presents the PCA clustering of guinea pigs according to digestibility variables, feeding levels (maintenance [M], restricted [R], and *ad libitum* [A]), and age groups. The vector directions indicate the gradient of increasing variable values, whereas vector lengths reflect their contribution to PC1 and PC2. Angles between vectors represent degrees of correlation among variables. Together, PC1 and PC2 explained 66.6% of the total variance. Animals fed at the maintenance level clustered mainly in the second and third quadrants, whereas *ad libitum*-fed animals were distributed in the first and fourth quadrants. Restricted-fed animals were spread across all quadrants, with a slight tendency toward quadrants 1 and 4.

### Hierarchical clustering analysis

**Figure 3:**
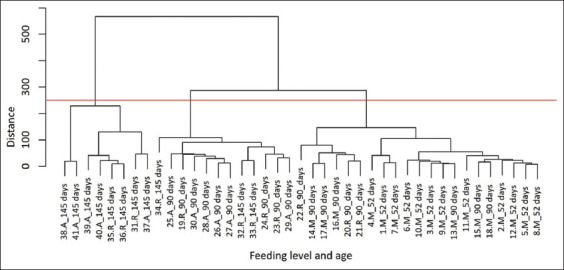
Euclidean clustering based on energy and crude protein digestibility variables, feeding levels, and age in Peruvian guinea pigs. The red line separates groups with similar characteristics. A_145, 145 days age *ad libitum*; A_90, 90 days age *ad libitum*; R_145, 145 days age restricted; R_90, 90 days age restricted; M_90, 90 days age maintenance; M_52, 52 days age maintenance.

Figure 3 depicts the hierarchical clustering of guinea pigs based on digestibility variables, age, and feeding levels. The vertical axis represents Euclidean distance, which quantifies similarity among individuals or groups. The red line delineates three distinct clusters:

- Group 1 included 145-day-old animals fed under *ad libitum* and restricted conditions
- Group 2 comprised mainly 90-day-old animals (75%) and some 145-day-old animals (25%), also under *ad libitum* and restricted feeding
- Group 3 contained all guinea pigs fed at the maintenance level (52 and 90 days), along with three 90-day-old individuals under restricted feeding.

## DISCUSSION

### Feed intake, age, and physiological growth

The increased DMI, energy, and CPI observed with less restrictive feeding and advanced age are consistent with the principles of physiological growth, in which nutrient demands rise for tissue development and the maintenance of larger body mass [8, 21, 22]. Strong positive correlations (r > 0.7) were identified between BW, absolute intake variables (DMI, GEI, MEI, CPI, and RP), and fecal output of crude protein, dry matter, and GE. Intake expressed as a percentage of BW decreased in older animals fed *ad libitum* (4.3% with a BW of 1142.9 g) compared to younger animals under maintenance feeding (5.5% with an LW of 396.1 g). Similar findings were reported by Tapie *et al*. [4], who observed relative intake increasing from 4.8% to 6.1% with age and weight gain. These results indicate an inverse relationship between BW, age, and relative feed intake. Such patterns are explained by chemostatic feedback mechanisms linked to energy density, metabolic end products, and gastrointestinal filling as animals approach physiological maturity [23–25], which limit overconsumption and maintain metabolic balance. Nonetheless, *ad libitum* feeding strategies in meat production are designed to maximize intake until market weight is achieved.

### Multivariate analysis of intake and efficiency

Multivariate analysis demonstrated that animals with higher PC1 scores, positioned in quadrants 1 and 4 of Figure 2, showed strong positive loadings for intake and BW variables (BW, DMI, GEI, MEI, CPI, fecal DM, and GEF) compared to animals at the maintenance level located in quadrants 2 and 3. PC2 distinguished digestibility and efficiency variables–such as DMD, DE, MEI/GEI, and RP/CPI (positive loadings)–from indicators of energy loss, including GEF/GEI, fecal CP/CPI, and urinary GE/GEI (negative loadings). Clustering by PC1 and PC2 indicated that guinea pigs fed *ad libitum*, especially at 145 days of age, exhibited high intake but reduced protein metabolic efficiency (DP/CPI: 75.4%) compared with maintenance-fed animals (DP/CPI: 84.5%). Comparable results for crude PD have been reported, with values declining from 84.5% (maintenance) to 81.9% (restricted) and 74.5% (*ad libitum*) [8].

### Digestibility and energy utilization

DMD peaked at 82.3% in guinea pigs fed at maintenance levels, compared with a minimum of 78.1% in those fed *ad libitum*. This pattern, where reduced intake improves digestibility, corroborates previous findings [4]. Hidalgo and Valerio [26] explained that lower intake extends gastrointestinal retention time, thereby enhancing enzymatic exposure and nutrient absorption. Similarly, energy metabolizability (ME/GE) remained high in restricted-fed animals but slightly decreased in older (145-day) *ad libitum*-fed guinea pigs, possibly due to faster intestinal transit reducing digestive and fermentative efficiency [6, 13].

### Protein utilization and retention efficiency

The MEI/GEI ratio peaked at 79.5% in animals fed *ad libitum* at 90 days, whereas other groups averaged ~77%. Animals under *ad libitum* and restricted feeding displayed reduced digestible protein (DP) and lower protein retention efficiency (RP/CPI) compared to maintenance-fed animals. This suggests that protein intake in high-intake groups surpassed the animals’ capacity for lean tissue deposition [27]. Similar trends were observed in rabbits by Lv *et al*. [28], where increasing dietary protein from 12% to 20% did not improve RP but instead elevated CP losses through feces and urine, lowering overall utilization efficiency.

At the maintenance intake level, reduced urinary and fecal CP was associated with a higher RP/CPI ratio, reflecting improved protein use under restricted feeding. Moon [29] also noted that guinea pigs exhibit higher metabolic efficiency during fasting compared to other rodents. When protein intake exceeds requirements, surplus amino acids are catabolized, increasing nitrogen excretion through feces and urine, thereby reducing nitrogen retention efficiency [21, 30–32].

### Implications of protein-energy balance

These results underscore the importance of maintaining a balanced protein–energy ratio, particularly in *ad libitum* regimens designed for rapid growth. The elevated fecal CP content observed in such systems reinforces this point. Nevertheless, the high post-absorptive metabolic efficiency (RP/DP > 96%) suggests that guinea pigs have a greater capacity to utilize dietary protein than other monogastrics, due to their digestive physiology, which combines enzymatic digestion in the stomach with microbial fermentation in the cecum and colon [33]. Given that protein is the most costly dietary nutrient, excessive intake not only reduces efficiency but can also strain renal function and increase environmental nitrogen pollution, as observed in rabbits [28, 34]. Thus, defining precise protein requirements for guinea pigs and rabbits is critical for optimizing feeding strategies in regions such as Bolivia, Peru, Ecuador, Colombia, Mexico, and parts of Africa where their meat is consumed [34–36].

### Role of multivariate analysis and study limitations

Traditional univariate methods for predicting digestibility and metabolism often fail to capture the complexity of biological interactions [4, 7]. In this study, cluster analysis (Figure 3) identified three distinct groups by age and feeding level, indicating that both factors significantly affect digestion and metabolism in guinea pigs raised for meat. These findings contrast with Tapie *et al*. [4, 8], who reported no effect of feeding level on DMD, DE, metabolic rate, or DP. This highlights the strength of multivariate analysis, which provides a more integrated understanding of physiological and nutritional interactions and supports the formulation of diets tailored to age and feeding level for optimized nutrient use and production efficiency.

A limitation of this study was the exclusive use of male Peruvian breed guinea pigs, which restricts generalization to females or other genetic lines. Nutrient digestibility and requirements vary with diet composition, physiological stage, sex, and breed [9, 10]. In addition, while the 6-day trial period may seem short, prior research has shown that 6–7 days is sufficient to obtain reliable digestibility estimates in guinea pigs [4, 7].

## CONCLUSION

This study demonstrated that feeding level and age significantly influence nutrient utilization efficiency in Peruvian guinea pigs. Higher intake under *ad libitum* feeding increased DMI, GEI, and CPI, but reduced protein retention efficiency (RP/CPI), which declined from 83.5% in maintenance-fed animals to 73.6% in the highest intake group at 145 days. In contrast, maintenance feeding achieved the highest digestibility values for dry matter (82.3%), DE (81.5%–82.1%), and protein utilization efficiency (DP/CPI: 84.5%). Energy metabolizability (ME/GE) peaked under *ad libitum* conditions (79.5% at 90 days), indicating that while energy use was enhanced, protein metabolism efficiency declined as intake exceeded the animals’ physiological requirements. PCA and clustering confirmed distinct metabolic groupings according to age and feeding regime.

These findings highlight the importance of adjusting the protein-energy ratio in guinea pig diets to balance growth performance with nutrient utilization efficiency. For production systems in the Andean region and Africa, formulating diets that avoid excessive crude protein supply can improve feed efficiency, reduce production costs, and minimize nitrogen excretion, thereby lowering environmental impacts. A major strength of this study was the application of multivariate tools, which provided a holistic perspective of nutrient partitioning and metabolic efficiency, moving beyond conventional univariate methods and enabling the identification of feeding-age patterns critical for designing tailored diets.

Nevertheless, the study was limited by its focus on male Peruvian breed guinea pigs, which constrains generalization to females and other genetic lines. Although the 6-day trial period may seem short, previous evidence supports its reliability for digestibility assessment. Future research should incorporate both sexes and multiple genetic lines, extend the trial duration, and include parameters such as carcass composition, reproductive performance, and environmental nitrogen assessments to strengthen recommendations. Modeling approaches integrating intake, metabolism, and growth dynamics could further refine feeding guidelines.

In conclusion, while *ad libitum* feeding maximizes growth and energy utilization, it compromises protein efficiency and elevates nitrogen losses. Conversely, maintenance-level feeding optimizes protein metabolism but limits growth potential. Balancing these trade-offs through stage-specific, protein–energy adjusted diets provides a promising strategy to improve productivity, enhance farmer profitability, and promote environmental sustainability in guinea pig production systems.

## DATA AVAILABILITY

The supplementary data can be made available by the corresponding author on request.

## AUTHORS’ CONTRIBUTIONS

WAT, CSER, and JFMH: Conceptualization, investigation, methodology, validation, writing - original draft, formal analysis, and data curation. WAT and CSER: Project administration and supervision. All authors have read and approved the final manuscript.
